# Two occurrences of delayed epidural hematoma in different areas following decompressive craniectomy for acute subdural hematoma in a single patient: a case report

**DOI:** 10.1186/s12893-017-0303-1

**Published:** 2017-12-04

**Authors:** Ruhong Wu, Jia Shi, Jiachao Cao, Yumin Mao, Bo Dong

**Affiliations:** Department of Neurosurgery, The Third Affiliated Hospital of Soochow University, Changzhou City, 213003 China

**Keywords:** Acute subdural hematoma, Acute epidural hematoma, Delayed epidural hematoma, Decompressive craniectomy, Computed tomography

## Abstract

**Background:**

Delayed epidural hematoma (DEH) following evacuation of traumatic acute subdural hematoma (ASDH) or acute epidural hematoma (EDH) is a rare but devastating complication, especially when it occurs sequentially in a single patient.

**Case presentation:**

A 19-year-old man who developed contralateral DEH following craniotomy for evacuation of a traumatic right-side ASDH and then developed a left-side DEH of the posterior cranial fossa after craniotomy for evacuation of the contralateral DEH. He was immediately returned to the operating room for additional surgeries and his neurological outcome was satisfactory.

**Conclusions:**

Although DEH occurring after evacuation of ASDH or acute EDH is a rare event, timely recognition is critical to prognosis.

## Background

Although delayed epidural hematoma (DEH) is an uncommon complication following evacuation of intracranial hematomas, it is devastating nevertheless, especially following the evacuation of traumatic acute subdural hematoma (ASDH). A previous study reported the incidence of delayed postoperative epidural hematoma (EDH) to be 1.0% [1]. A total of 38 cases of such DEH were found in literature (Table 1). We report a case involving a Chinese man who developed contralateral DEH following decompressive surgery for right-side ASDH, and then developed an additional remote DEH following decompressive surgery for the contralateral DEH.

**Table 1:** Cases of DEH, found in literature

	Authors	Age/Sex	GCS	GOS
1	Xu et al. [16]	10/F	6	5
2	Meguins et al. [15]	39/M	6	1
3	Nadig et al. [17]	21/M	3	5
4	Shen et al. [18]	51/M	4	5
5		35/M	3	1
6		43/M	4	1
7		52/M	5	4
8		40/F	3	2
9	Saberi et al. [5]	19/M	6	2
10	Su et al. [9]	39/M	6	3
11		70/M	9	5
12		35/F	5	1
13		43/F	9	5
14		40/M	4	2
15		38/M	4	2
16		19/M	9	5
17		25/F	4	1
18		44/M	4	1
19		28/M	5	2
20		25/F	4	2
21		19/F	6	1
22	Mohindra et al. [11]	45/M	7	4
23		28/M	4	1
24	Boviatsis et al. [13]	49/M	8	1
25	Cohen et al. [19]	76/F	7	5
26	Matsuno et al. [20]	17/M	3	4
27		31/M	6	4
28		40/M	3	3
29		31/M	4	4
30	Feuerman et al. [21]	29/F	6	2
31		18/M	9	3
32		16/F	4	1
33	Meguro et al. [22]	22/M	5	3
34		54/F	4	1
35	Borovich et al. [23]	39/M	4	1
36	Piepmeier et al. [24]	39/M	3	3
37		23/M	7	3
38		24/M	6	2

*GCS* Glasgow Coma Scale, *GOS* Glasgow Outcome Scale

## Case presentation

**Fig. 1:**
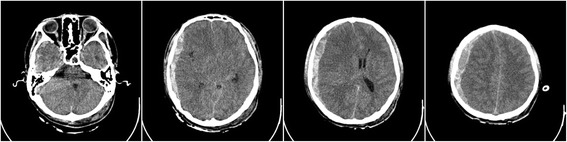
Admission computed tomography showing right acute subdural hematoma with midline shift

**Fig. 2:**
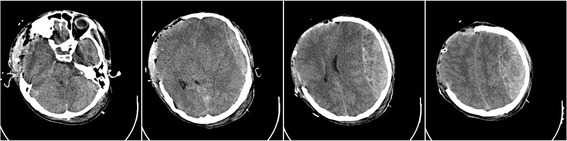
Computed tomography following the first decompressive surgery showing contralateral delayed epidural hematoma

**Fig. 3:**
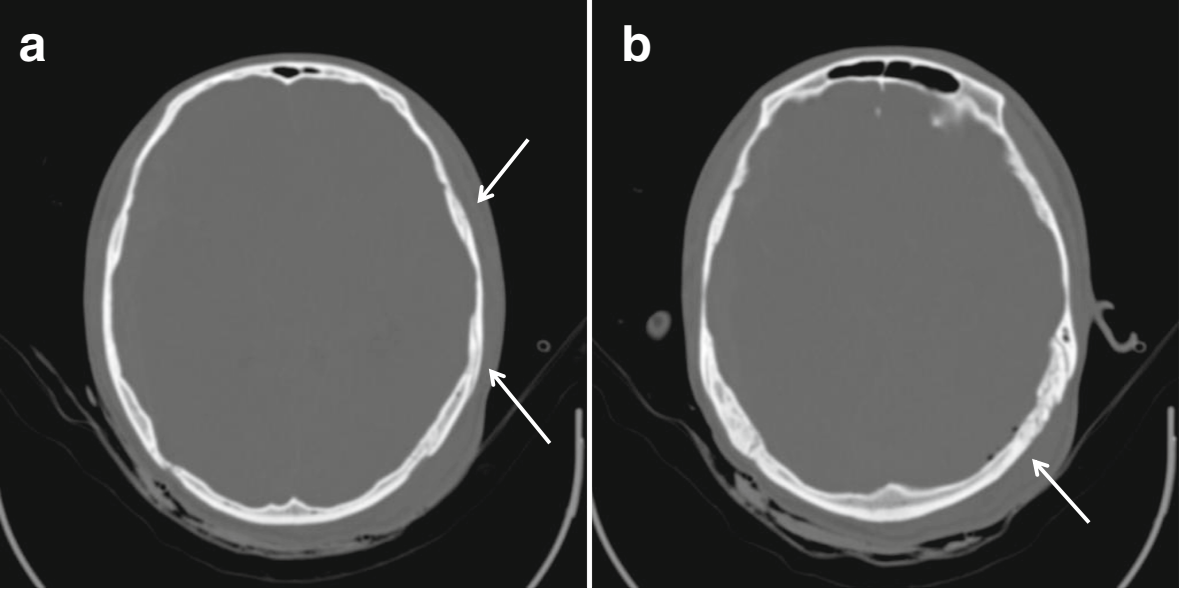
**a** The Axial Bone window showing a left temporal bone fractures. **b** The Axial Bone window showing left occipital intracranial pneumatocele

A 19-year-old Chinese man involved in a motor vehicle accident was admitted to the emergency department presenting with a Glasgow Coma Scale (GCS) of 3 and right eye mydriasis. A computed tomography (CT) scan of his brain revealed right-side ASDH and a midline shift of 14.5 mm with severe brain swelling (Figs. 1 and 3). He was hemodynamically stable, with no clotting dysfunction according laboratory tests. He was subsequently transferred to the operating room and underwent a right decompressive craniectomy. The ASDH was caused by a ruptured lateral fissure vein. The brain exhibited slowly progressing swelling after the hematoma and inactivated brain tissue were evacuated. Consequently, immediate augmentation duraplasty using artificial dura mater was performed. On examination, the patient exhibited bilateral mydriasis. Given the slowly progressing brain swelling, this was possibly due to delayed contralateral ASDH or EDH. A CT scan of the brain was performed immediately thereafter, which revealed the emergence of contralateral DEH (Fig. 2). Owing to a mass effect from the DEH and severe brain swelling, the patient was immediately taken to the operating room to undergo a left hematoma evacuation and decompressive craniectomy. During surgery, it was determined that the DEH was caused by the rupture of the middle meningeal artery branch, with temporal and occipital bone fractures (Fig. 3a). Following surgery, the patient was transferred to the neurological intensive care unit, where the left and right pupil sizes were measured to be 2.0 mm and 5.0 mm, respectively.

**Fig. 4:**
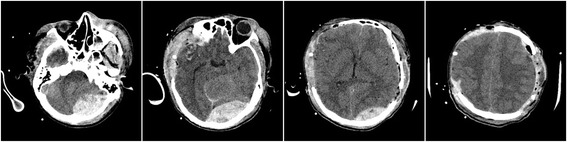
Computed tomography following the second decompressive surgery showing remote delayed epidural hematoma

**Fig. 5:**
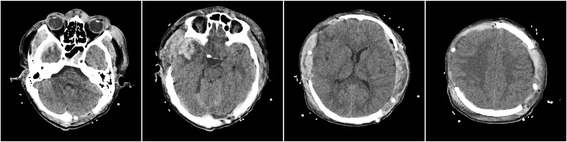
Computed tomography following the third decompressive surgery

On postoperative day 1, approximately 5 h after the second surgery, the primary nurse found that the size of the patient’s left pupil gradually increased from 2.0 mm to 3.5 mm. An immediate brain CT scan revealed evidence of left-side DEH of the posterior cranial fossa (Fig. 4). Owing to mass effect of the DEH, a third surgery was offered. He underwent left posterior cranial fossa hematoma evacuation and decompressive craniectomy. During the third surgery, it was determined that the DEH was caused by a ruptured transverse sinus. A brain CT following the third surgery was performed (Fig. 5). The patient recovered to a GCS of 7 within 40 days after surgery, and was transferred to the rehabilitation hospital. He was ultimately discharged from the rehabilitation hospital with a Glasgow Outcome Score of 4. He underwent cranioplasty 1 year later and has since recovered well.

## Discussion and conclusion

Traumatic brain injury (TBI) is the leading cause of morbidity and mortality in adults in highly developed countries, with the highest incidence in men aged 15–24 years [2]. Approximately one-third of patients with severe TBI are diagnosed with ASDH [3]. According to the guidelines for severe TBIs, ASDH causing brain herniation should be immediately evacuated with or without bone flap removal and duraplasty [4]. DEH following decompressive surgery for ASDH or acute EDH is an extremely uncommon occurrence, with only dozens of cases in the medical literature published in English (Table 1); nevertheless, it is a devastating complication [5–8]. To our knowledge, our report is the first to document two occurrences of DEH in different areas following decompressive craniectomy for traumatic ASDH in a single patient, who developed DEH sequentially following decompressive surgery for ASDH and DEH.

There were some signs that alerted us to the possibility of DEH following evacuation of the ASDH or acute EDH, including a skull fracture, intraoperative brain swelling, pupillary dilation, an unmanageable elevated intracranial pressure (ICP) [9], a large volume of intraoperative blood loss, long duration of craniotomy, and a large craniotomy area [10]. During the first surgery in this case, the brain exhibited slowly progressing swelling, with bilateral mydriasis after the hematoma and inactivated brain tissue were evacuated. A subsequent CT scan revealed a large contralateral DEH. On postoperative day 1 after the second surgery, the size of the patient’s left pupil gradually increased from 2.0 mm to 3.5 mm, with a CT scan revealing a large posterior fossa DEH. When these alarm signs presented, we need an immediate brain CT scan to exclude remote DEH.

There are many explanations for the pathogenesis of DEH following evacuation of ASDH or acute EDH, including loss of tamponade effect, abnormal vasomotor mechanisms, and acute coagulopathy [11]. The main cause, however, appears to be a disruption of the equilibrium in damaged vessels and reactive intracranial hypertension [12]. Sources of bleeding include a ruptured meningeal arterial branch or a skull fracture [13, 14]. We believe that the primary mechanism of DEH in this case was the loss of the tamponade effect with the rupture of the meningeal arterial branch and transverse sinus, and skull fractures. Thus, if there is a possibility of remote DEH after surgery, a CT scan of the brain is necessary. In this case, the brain exhibited slowly progressive swelling during the first surgery with the left temporal and occipital bone fractures, a postoperative brain CT would been taken immediately to exclude the possibility of delayed hematoma; meanwhile, the patient’s family were struggling financially, and the imported ICP monitor was cost prohibitive. Consequently, an ICP monitor was not implanted. But if it was feasible, implantation of a continuous ICP monitor during the first surgery would have helped us to recognize the DEH in a timely manner [15].

This case suggests that DEH following craniotomy for evacuation of traumatic ASDH or acute EDH should always be considered a possibility, especially in cases of intra-operative brain swelling and skull fractures, even though CT may provide no evidence of remote skull fractures. Cautious observation and early postoperative radiological evaluation may facilitate timely recognition of these remote DEHs and contribute to improved patient outcomes.

## Abbreviations

ASDH: Acute subdural hematoma
CT: Computed tomography
DEH: Delayed epidural hematoma
EDH: Epidural hematoma
GCS: Glasgow coma scale
GOS: Glasgow outcome scale
ICP: intracranial pressure
TBI: Traumatic brain injury
